# *GABRB1* Single Nucleotide Polymorphism Associated with Altered Brain Responses (but not Performance) during Measures of Impulsivity and Reward Sensitivity in Human Adolescents

**DOI:** 10.3389/fnbeh.2017.00024

**Published:** 2017-02-15

**Authors:** Theodora Duka, Kyriaki Nikolaou, Sarah L. King, Tobias Banaschewski, Arun L. W. Bokde, Christian Büchel, Fabiana M. Carvalho, Patricia J. Conrod, Herta Flor, Jürgen Gallinat, Hugh Garavan, Andreas Heinz, Tianye Jia, Penny Gowland, Jean-Luc Martinot, Tomáš Paus, Marcella Rietschel, Trevor W. Robbins, Michael Smolka, Gunter Schumann, David N. Stephens

**Affiliations:** ^1^School of Psychology, University of SussexFalmer, UK; ^2^Central Institute of Mental Health, Medical Faculty Mannheim/Heidelberg UniversityMannheim, Germany; ^3^Institute of Neuroscience, Trinity College DublinDublin, Ireland; ^4^Department of Systems Neuroscience, Universitätsklinikum Hamburg EppendorfHamburg, Germany; ^5^Institute of Psychiatry, Kings College LondonLondon, UK; ^6^Department of Psychiatry, Université de Montréal, CHU Ste Justine HospitalMontréal, QC, Canada; ^7^Departments of Psychiatry and Psychology, University of VermontBurlington, VT, USA; ^8^Clinic for Psychiatry and Psychotherapy, Charité UniversitätsmedizinBerlin, Germany; ^9^School of Psychology, University of NottinghamNottingham, UK; ^10^INSERM, UMR 1000, Research Unit Imaging and Psychiatry, IFR49, CEA, DSV, I2BM-Service Hospitalier Frédéric JoliotOrsay, France; ^11^School of Psychology, University of NottinghamNottingham, UK; ^12^Rotman Research Institute, University of TorontoToronto, ON, Canada; ^13^Department of Psychology, University of CambridgeCambridge, UK; ^14^Department of Psychiatry and Psychotherapy, Technische Universität DresdenDresden, Germany; ^15^MRC Social, Genetic and Developmental Psychiatry (SGDP) CentreLondon, UK

**Keywords:** alcohol abuse, stop signal, monetary incentive delay, fMRI, GABA_A_ receptor, inferior frontal gyrus, insula, supramarginal gyrus

## Abstract

Variations in genes encoding several GABA_A_ receptors have been associated with human drug and alcohol abuse. Among these, a number of human studies have suggested an association between *GABRB1*, the gene encoding GABA_A_ receptor β1 subunits, with Alcohol dependence (AD), both on its own and comorbid with other substance dependence and psychiatric illnesses. In the present study, we hypothesized that the *GABRB1* genetically-associated increased risk for developing alcoholism may be associated with impaired behavioral control and altered sensitivity to reward, as a consequence of altered brain function. Exploiting the IMAGEN database (Schumann et al., 2010), we explored in a human adolescent population whether possession of the minor (T) variant of the single nucleotide polymorphism (SNP) rs2044081 is associated with performance of tasks measuring aspects of impulsivity, and reward sensitivity that are implicated in drug and alcohol abuse. Allelic variation did not associate with altered performance in either a stop-signal task (SST), measuring one aspect of impulsivity, or a monetary incentive delay (MID) task assessing reward anticipation. However, increased functional magnetic resonance imaging (fMRI) blood-oxygen-level dependent (BOLD) response in the right hemisphere inferior frontal gyrus (IFG), left hemisphere caudate/insula and left hemisphere inferior temporal gyrus (ITG) during MID performance was higher in the minor (T) allelic group. In contrast, during SST performance, the BOLD response found in the right hemisphere supramarginal gyrus, right hemisphere lingual and left hemisphere inferior parietal gyrus indicated reduced responses in the minor genotype. We suggest that β1-containing GABA_A_ receptors may play a role in excitability of brain regions important in controlling reward-related behavior, which may contribute to susceptibility to addictive behavior.

## Introduction

Alcohol dependence (AD) is a complex, heterogeneous disease with both strong genetic and environmental influences in its etiology. Heritability estimates for the susceptibility for AD explain between 50% and 60% of variance (Stacey et al., 2009). Recently, a number of genes encoding subunits of GABA_A_ receptors have been associated with both AD and addiction to other drugs (for a review see Stephens et al., 2017).

Across mammalian species, genes encoding many of the GABA_A_ subunits are organized into chromosomal clusters. In humans, *GABRA2, GABRA4, GABRB1* and *GABRG1*, encoding for α2, α4, β1, γ1 subunits, respectively, are localized on chromosome 4p12 (Song et al., 2003). Gene association studies have consistently identified single nucleotide polymorphisms (SNPs) and haplotypes in this region to be associated with both alcohol and other drug addictions. Variations in *GABRA2* have been most frequently associated with addictions and related behaviors (Covault et al., 2004; Edenberg et al., 2004; Lappalainen et al., 2005; Dixon et al., 2010; Enoch et al., 2010), but there is also a robust association of *GABRB1* with AD comorbid with other substance dependence and psychiatric illnesses (Kertes et al., 2011; Yang et al., 2012). Interestingly, the strength of the association with AD alone is less clear (Parsian and Zhang, 1999; Dick and Foroud, 2003; Song et al., 2003; Reck et al., 2005). Very recently, an association has been identified between the intergenic SNP rs2044081 in *GABRB1* and AD in a large (611 cases, 646 controls), well characterized British/Irish population (Odds Ratio 4.2 (95% Confidence Intervals 1.5–11.5) *P*_corrected_ 3.31 × 10^2^; McCabe et al., 2017).

While gene association data may suggest the contribution of the gene to the condition studied, they do not provide information as to how the gene contributes to the phenotype. GABA_A_ receptors play a crucial role in circuitries important in addiction processes, and genetic variations may elicit a change in function of brain areas underlying behavioral traits such as impulsivity and reward sensitivity that predispose to addiction. We were therefore interested to discover whether variations in SNP rs2044081 of *GABRB1* associated with risk for AD, also predisposed to impulsive behavior, and altered sensitivity to reward. However, impulsivity is exacerbated by drug use (Hogarth, 2011). Thus, in order to assess genetic associations of *GABRB1* variants with impulsivity, it was important to study such associations prior to the development of alcohol abuse. For this reason, it was particularly informative to study genetic associations with brain functionality during performance of tasks measuring impulsivity and reward sensitivity in adolescence, before AD develops. For this purpose, we used data collected within the IMAGEN study of adolescents (Schumann et al., 2010). Besides measurements of alcohol use we have also acquired measurements of drug taking and smoking habits. As alcohol abuse is associated with stress in early life (Stephens et al., 2017), we also included data obtained from a life event questionnaire.

In the current article we examine the association of variants in this SNP with variations in behavioral measures associated with vulnerability to alcohol abuse, and in blood-oxygen-level dependent (BOLD) contrast imaging, using functional magnetic resonance imaging (fMRI) in adolescents. We thus exploited the IMAGEN database (Schumann et al., 2010) to identify individuals carrying the major and minor alleles of the rs2044081 SNP in a population of 14-year olds, and investigated performance in tests of reward sensitivity and impulsivity, and brain responses, using fMRI, during the performance of these tasks. There is emerging evidence that individuals with alcohol dependency have a decreased sensitivity to rewards (which correlates with hypoactivity in the nucleus accumbens (NAc; Volkow et al., 2010). It has been postulated that this hypoactivity leads to drug use to compensate for the deficit, and in turn disrupts metabolism of various prefrontal regions to increase impulsivity and to lead to drug taking becoming compulsive and habitual (Hogarth, 2011).

Both subcortical (Li et al., 2008) and, more consistently, cortical prefrontal regions such as orbitofrontal cortex, anterior cingulate cortex (ACC) and inferior frontal gyrus (IFG) show hypoactivity during performance of a stop-signal task (SST) in people who have used illicit substances or are predisposed to substance dependence (Whelan et al., 2012; Nymberg et al., 2013a), while prefrontal cortex (PFC) reduced activation correlates negatively with performance. In the monetary incentive delay (MID) task, in healthy adolescent volunteers, reward sensitivity is associated with activation of the ventral striatum during anticipation of the reward (Knutson et al., 2000; Nees et al., 2012a,b). However, in adolescents with problematic substance use, and in individuals predisposed to substance dependence, hypoactivity in the NAc was found during performance in tasks involving reward sensitivity measurements (Andrews et al., 2011; Peters et al., 2011; Schneider et al., 2012).

Therefore, the aim of the present study is to investigate the influence of the rs2044081 gene variant on reward sensitivity and impulsivity in adolescents. It is hypothesized that: (1) individuals carrying the minor (T) allele will have lower BOLD responses in the prefrontal regions during SST which will correlate with impaired performance; and (2) individuals carrying the minor allele will show lower responses in the NAc during MID which will correlate with impaired performance.

## Materials and Methods

### Participants

Pre-existing data collected from 1299 participants under the IMAGEN project were used (details of the IMAGEN project’s study design, recruitment procedures, inclusion/exclusion criteria and data storage/safety information can be found in Schumann et al., 2010) to test a hypothesis that variations in the rs2044081 SNP of *GABRB1* are associated with altered brain activity during performance of tasks implicated in the development of addictive behavior. Generally serious medical conditions (e.g., diabetes, rheumatologic disorders, neurological or developmental conditions), previous trauma with loss of consciousness, MRI contraindications (e.g., metal implants and claustrophobia) or adolescents with IQ <70 were exclusion criteria. Participants were also excluded if their genotyping, neuroimaging, or behavioral data did not pass the IMAGEN project’s quality control checks. There were 627 males and 672 females in the sample. 1144 were right handed and 155 were left handed or ambidextrous. Participants were 14 years old at time of data collection and were tested at eight IMAGEN assessment centers (London, Nottingham, Dublin, Mannheim, Dresden, Berlin, Hamburg, and Paris). Ethical approval was provided by the local ethical committees of each assessment center, and these procedures have been described previously (see Schumann et al. (2010) for a list of the assessment centers involved). All variables were studied across all locations using a standardized procedure across centers. Written informed consent was obtained from a parent or guardian, and verbal assent was obtained from the adolescent. Any adolescents with IQ <70 were excluded from this study.

### Design

Participants were allocated to allelic groups depending on the presence or absence of the minor T allele of rs2044081. Each participant was identified as being either homozygous for the minor allele, homozygous for the major allele, or heterozygous. A between subjects design was used. The independent variable was the allelic group for the SNP and comprised three levels: homozygous minor (*N* = 30; 11 male), heterozygous (*N* = 305; 138 male) and homozygous major (*N* = 964; 479 male). For the subset of the 522 participants for whom data for the SST is available, the corresponding numbers were: homozygous minor (*N* = 10; 5 male), heterozygous (*N* = 116; 53 male) and homozygous major (*N* = 396; 183 male).

### Materials

#### Stop-Signal Task (SST)

**Figure 1:**
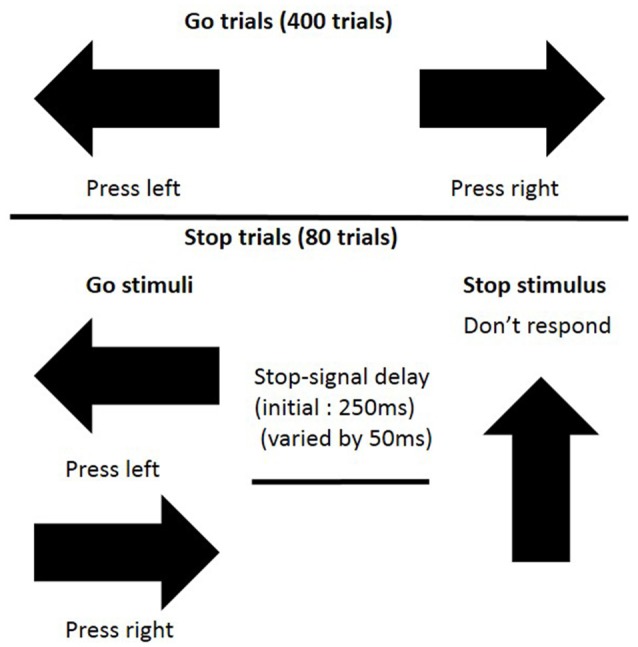
**Schematic display of stop-signal task (SST) procedure (cited in Rubia et al., 2001)**.

On each trial of the SST (see Figure 1 for a schematic outline), an arrow (go signal), that pointed either to the left or to the right, was presented in the center of the computer screen. Participants were asked to indicate the direction of the arrow by pressing one of two buttons as quickly and as accurately as they could. On 20% of the trials (80 trials), the go signal was followed by a stop signal (an arrow pointing upwards), and participants were told that in those instances, they should refrain from responding. Stopping difficulty was manipulated across trials by varying the onset of the stop signal after the go signal (stop-signal delay), using an algorithm which has been previously described (Rubia et al., 2001), so that participants successfully stopped on 50% of trials. A block contained 400 go trials with a stimulus duration of 1000 ms, and 80 stop trials with a stimulus duration of 0–900 ms (50 ms steps; initial delay 250 ms) in accordance to the algorithm.

The main outcome variable was stop signal reaction time (SSRT), which was calculated by subtracting the mean stop-signal delay from the Go RT at the percentile corresponding to the proportion of unsuccessfully inhibited stop trials. Participants were familiarized with the task prior to scanning by performing 60 trials in a 2 min practice session. Due to technical problems with calculating the latency referring to the ability to successfully stop the initiated response in the SST, some participants’ SST data were unusable. Thus data collected only from a subset of 522 participants (241 males and 281 females; 461 were right handed, and 61 were left handed or ambidextrous) are presented with regard to performance on SST.

#### Monetary Incentive Delay Task (MID; Knutson et al., 2000)

**Figure 2:**
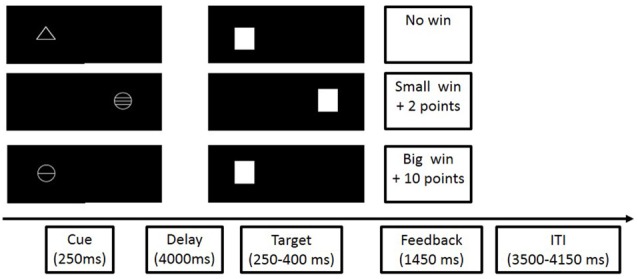
**Schematic outline of the stages of monetary incentive delay (MID; cited in Nymberg et al., 2013a)**.

On each trial of the MID task (see Figure 2 for a schematic outline), one of three cues (a triangle; a circle with a line though it; or a circle with three lines through it), was presented for 250 ms, either to the left or to the right of the screen. The type of cue, and the cue’s location predicted the reward value (possibility of winning 0, 2, or 10 points upon correct responding), and the location (left or right side of the screen), respectively, of a subsequently presented target stimulus (a white square). The cue was followed by a fixation cross (4500 ms anticipation period), which in turn was followed by the presentation of the target stimulus for a varied duration (250–400 ms). Participants were told that they could win the predicted reward if they correctly indicated the location of the target, by pressing a button with the index finger of either their left or their right hand. If participants responded too early or too late they did not receive points. Feedback on reward points was given following the presentation of the target stimulus, and in order to increase motivation, participants received a single M&M sweet for every five points that they won. Task difficulty was varied using a tracking algorithm that ensured that participants were successful on 66% of trials, and did not win more than 200 points. There were 22 trials per condition (no win, small win, big win), and total task duration was 11 min.

Participants were familiarized with the task prior to scanning by performing a practice session for 3 min. While in the scanner, participants were reminded of the instructions. The outcome measure of the MID task was the difference score between the frequency of successful hits in big win trials and the frequency of successful hits during no win trials (MID-Diff). The higher the difference score, the higher was the frequency of responding correctly on trials on which a high reward was anticipated.

#### Questionnaires

The Alcohol Use Disorders Identification Test (AUDIT; Saunders et al., 1993) is designed to identify individuals with harmful or hazardous alcohol consumption, and was used to measure history and severity of alcohol use. It consists of 10 questions measuring alcohol use history, and an individual’s assessment of other’s feelings towards their alcohol consumption. The present study used the total AUDIT score (AUDIT-Total) in analyses, with high scores reflecting high severity of alcohol use. Additionally, individual reports on number of drinking occasions were noted (see Table 1).

**Table 1:** **Sample characteristics (gender and handedness distribution Alcohol Use Disorders Identification Test (AUDIT)-Total, Life Events Questionnaire (LEQ)-Total scores, Puberty development score and drinking habits), and behavioral data (proportion of correct responses to large and no wins as well as differences of large win no win in the monetary incentive delay, MID (MID-diff); stop signal reaction time (SSRT) and RT of correct go responses in the stop-signal task (SST))**.

SNP rs2044081	Homozygous minor (*n* = 30; male = 11)	Heterozygous (*n* = 305; male = 137)	Homozygous major (*n* = 964; male = 479)
**Handedness (N)**
Right	25	267	852
Left	5	36	104
Both	0	2	8
AUDIT-Total	1.33 (2.20)	1.33 (2.10)	1.51 (2.61)
LEQ-Total	14.83 (4.81)	14.01 (4.82)	14.36 (4.45)
PDS score female	4.27 (0.70)	4.32 (0.69)	4.31 (0.71)
PDS score male	2.39 (0.40)	2.64 (0.57)	2.65 (0.51)
Occasions drinking in lifetime	1.80 (1.54)	2.02 (1.78)	1.98 (1.75)
Occasions drinking >5 drinks	1.67 (0.81)	1.95 (1.38)	1.79 (1.41)
MID correct large win (proportion)	70.30 (14.90)	66.85 (12.62)	67.36 (12.61)
MID correct no win (proportion)	49.70 (20.22)	51.74 (16.83)	51.10 (17.73)
MID-Diff (proportion)	20.61 (28.14)	15.11 (20.99)	16.25 (22.51)
SSRT (ms)	223.57 (27.67)	220.79 (37.57)	220.63 (38.7)
SS correct go RT (ms)	433.06 (52.78)	432.21 (55.98)	428.47 (62.81)

*Data are presented as Mean and Standard Deviation (SD) for each allelic group separately. PDS, Puberty Development Scale*.

The Life Events Questionnaire (LEQ; adapted from Newcomb et al., 1981) was used to measure the amount and degree of severity of stressful life events that occurred throughout the participant’s life. The questionnaire consists of 39 items that measure the occurrence (“ever” and “in the past year”), and the perceived affective impact (rated on a 5-point scale) of common early life events covering the following domains: Family/Parents, Accident/lllness, Sexuality, Autonomy, Deviance, Relocation and Distress. The present study used the total count of life-time events (LEQ-Total) in the analyses, with high scores reflecting a high number of stressful life events.

The Puberty Development Scale (PDS; Petersen et al., 1988), a self-report measure of physical development, with separate forms for males and females, was used to ascertain that male and female participants in allelic groups did not differ with respect to their physical development. Participants responded to questions about their growth in stature and pubic hair, as well as menarche in females and voice changes in males. An average score was calculated for each item.

### Procedures

#### Genotyping

DNA purification and genotyping was performed by the Centre National de Génotypage in Paris. DNA was extracted from whole blood samples preserved in ethylene-diamine-tetra-acetic acid (EDTA) vacutainer tubes (BD, Becton, Dickinson and Company, Oxford, UK) using Gentra Puregene Blood Kit (QIAGEN, Valencia, CA, USA) according to the manufacturer’s instructions. Genotype information was collected at 582, 982 markers using the Illumina HumanHap610 Genotyping BeadChip (Illumina, San Diego, CA, USA) as part of a previous genome wide association study (Schumann et al., 2010).

#### Functional Magnetic Resonance Imaging

##### MRI

Imaging data were acquired at eight IMAGEN assessment sites with 3T MRI scanners by several manufacturers (Siemens, Philips, General Electric, Bruker). Full details of the MRI acquisition protocols and quality checks have been described previously (Schumann et al., 2010). The same scanning protocol was used at all sites. In brief, for each participant, high-resolution anatomical images were acquired with a T1-weighted magnetization prepared gradient echo (MPRAGE) sequence.

Functional MRI images were acquired with an echo-planar imaging (EPI) sequence. For each participant, 300 volumes were acquired for the MID task, and 444 volumes were acquired for the SST. For both tasks, each volume consisted of 40 slices (2.4-mm slice thickness, 1-mm gap) and echo time was optimized (TE = 30 ms; TR = 2.2 s) to provide reliable imaging of subcortical areas.

### Data Analysis

Gender, handedness and IMAGEN center were included as covariates for all analyses, behavioral and imaging.

#### Behavioral

Differences between allelic groups on SST and MID indices (i.e., SSRT and MID-Diff, respectively) were determined using separate one-way ANCOVAs.

To determine the impact of life stress history on reward sensitivity and impulsivity, separate Bonferroni corrected correlations were performed on the relationship between LEQ-Total and: (a) SSRT; (b) MID-Diff; and (c) AUDIT-Total scores for each SNP’s allelic group.

#### fMRI

Functional MRI data were analyzed with SPM8 and Matlab (2011b). The pre-processing of the functional MRI data has been described previously (Nymberg et al., 2013b). Briefly, the data were slice-time corrected; all volumes were aligned to the first volume; and non-linear warping was performed to normalize slices to the standard Montreal Neurological Institute (MNI) space. Images were then smoothed with a Gaussian kernel of 5-mm full width at half-maximum (FWHM).

At the first level of analysis of the MID functional MRI data, linear models were created by convolving the canonical hemeodynamic response function with the onsets of the anticipation and feedback periods for each cue type (i.e., anticipation hit big win, anticipation hit small win, anticipation hit no win, anticipation missed big win, anticipation missed small win, anticipation missed no win, anticipation no response, feedback hit big win, feedback hit small win, feedback hit no win, feedback missed big win, feedback missed small win, feedback missed no win, press left, press right). For each participant movement parameters were added to the model as regressors of no interest. The contrast “anticipation big win vs. anticipation no win” (MID-contrast) was computed for each participant as an index of neural activity associated with anticipation of a large reward.

Similarly, at the first level of analysis, for the SST functional MRI data, for each participant, linear models were created by convolving the canonical hemeodynamic response function with the onsets of each trial-type (i.e., go success, go too late, go wrong, stop success and stop failure) to form regressors of interest. Movement parameters were added to the design matrix as regressors of no interest. The “stop success-go success” contrast (SST contrast) was computed for each participant in order to measure neural activity associated with successful stopping.

MID and SST contrasts were submitted to separate 2nd-level one-way ANCOVAs, with testing-site, gender and handedness included as regressors of no interest, to test for differences between allelic groups. The main effect of genotype (i.e., homozygotes minor vs. heterozygotes vs. homozygous majors) was computed as an F contrast thresholded at *p* = 0.005 and a cluster extent threshold of *k* = 22 voxels. This conjunction of specific voxel-level and cluster-extent thresholds corresponds to a whole-brain-corrected significance of *p* < 0.05.

The non-arbitrary cluster-extent threshold was determined by Monte-Carlo simulations using the same parameters as in our study (Green et al., 2009, 1000 iterations^1^; see Katanoda et al., 2002; Ross and Slotnick, 2008).

### Regressions

The coordinates of each significant cluster peak resulting from the factorial analyses (i.e., main effect of group in each ANCOVA) were used as centers of 4 mm sphere Regions-of-Interest (ROIs), created using MarsBaR^2^. For all participants, separate 2nd-level regression models tested significant relationships between regional activity resulting from the MID and SST contrasts within these ROIs and the Monetary Incentive Delay difference (MID-Diff) and SSRT, respectively. Additionally, these two contrasts were also entered into regression models with the AUDIT-Total scores in order to test whether BOLD responses associated with the anticipation of a large reward, or successful stopping was related with severity of alcohol use. For all regression models, F contrasts examining both positive and negative associations were computed and thresholded at *p* = 0.005 with a cluster extent threshold of *k* = 22 voxels.

## Results

### Sample Characteristics and Behavioral Results

Means and standard deviation (SDs) of AUDIT, drinking habits and LEQ score, as well as behavioral results are presented in Table 1. Gender and handedness distribution is also given in Table 1. Homogeneity of variance was not violated in any analysis (*F* > 0.75, *ns*).

Ethnicity information was missing from four participants in the entire sample, three of which were also participants that were included in the sub-group that additionally completed the SST.

Allelic groups were matched well on gender ratio (*χ*^2^ < 3.8, ns, in all cases), and neither the male nor the female participants differed in pubertal development among allelic groups (*F* < 1.4, ns, in both cases; see Table 1). Allelic groups consisted predominantly of individuals whose parents were both of Caucasian ethnicity (Minor: 28/29; Heterozygous: 285/303; Major: 863/963). Comparisons showed that the minor allelic group did not differ from either the heterozygous or the major groups in the distribution of ethnic background (*χ*^2^ < 1.5, ns, in both cases). However, a difference in ethnic background distribution was found between the heterozygous and major allele groups (*χ*^2^_=_ 5.39, *p* < 0.05).

From the subgroup that additionally completed the SST (*n* = 522), allelic groups were matched well on gender ratio (*χ*^2^ < 1, ns, in all cases), and neither the male nor the female participants differed in pubertal development among allelic groups (*F* < 1, ns, in both cases). As with the larger cohort, this subgroup also consisted predominantly of individuals whose parents were both of Caucasian ethnicity (Minor: 8/9; Heterozygous: 105/115; Major: 355/395). Comparisons showed no differences between allelic groups in the distribution of ethnic background (*χ*^2^ < 1.75, ns, in all cases).

The covariates included in the ANCOVAs did not correlate with the MID-Diff scores or SSRT.

After controlling for covariates, there were no differences between the allelic groups in MID-Diff, GO Reaction Time, or SSRT scores (all *F*s < 1, ns).

No effects of genotype was found for AUDIT or LEQ score (*F*_(2,1296)_ = 0.600, ns, and *F*_(2,1296)_ = 0.900, respectively). No significant correlations were revealed between LEQ-Total and: SSRT, MID-Diff and AUDIT-Total scores within each allelic group.

### Brain Imaging

#### Monetary Incentive Delay

**Figure 3:**
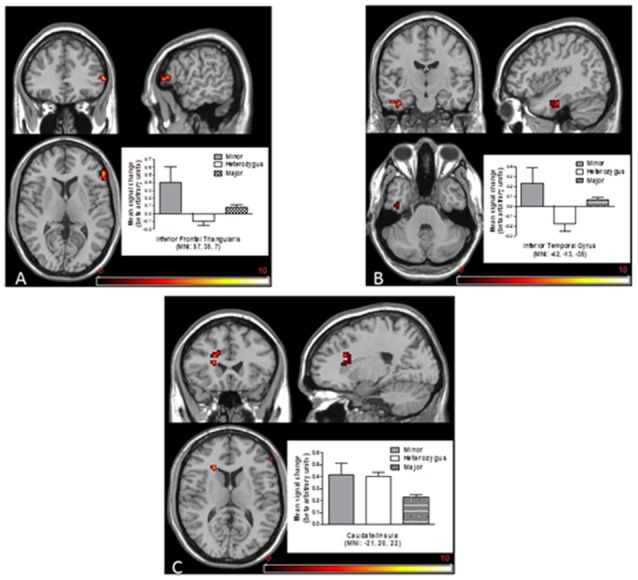
**Activity enhancement or reduction associated with large win vs. no win in MID during the anticipation phase in the group of homozygous minor, heterozygous and homozygous major for the SNP rs2044081.** Increased blood-oxygen-level dependent (BOLD) responses within **(A)** the right inferior frontal triangularis and **(B)** the left inferior frontal gyrus (IFG) was found only in the group of homozygous minor; also responses within **(C)** caudate/insula was larger in the homozygous minor group compared with the other two groups. Data are presented in mean ± SEM.

Despite the similarity in performance, there was a difference in BOLD response found in the right hemisphere IFG (*F*_(2,1293)_ = 7.75, *p* < 0.005), left hemisphere caudate/insula (*F*_(2,1293)_ = 7.69, *p* < 0.005) and left hemisphere inferior temporal gyrus (ITG; *F*_(2,1293)_ = 8.25, *p* < 0.005), with higher responses seen in the minor (TT) genotype. Contrasts between the groups revealed a significantly higher brain response in the minor group than either the major or the heterozygous group (*t*s > 1.7, *p*s < 0.01 in both cases, see Figure 3A), with regard to the IFG. Regarding ITG and the caudate, contrasts between the homozygous major and the heterozygous genotype were significant (*t*_(1267)_ = −0.3.17, *p* < 0.001 and *t*_(1267)_ = −0.3.87, *p* < 0.001, respectively; see Figures 3B,C). See Table 2 for details on brain areas. Caudate BOLD changes were different in males and females. A gender main effect (*F*_(1,1293)_ = 4.860, *p < 0.05*) but not a gender by genotype interaction (*F*_(2,1293)_ = 0.270, *ns*), was found. Males showed a higher BOLD signal compared to females.

**Table 2:** **Whole brain magnitude related *F* scores and Montreal Neurological Institute (MNI) coordinates of response peak for main effect of allelic group on the MID task**.

Region	Cluster	L/R	*F*	MNI coord (*x, y, z*)
Inferior temporal gyrus	26	L	8.25	(−42, −13, −35)
			6.02	(−48, −16, −29)
Inferior frontal triangularis	23	R	7.75	(57, 35, 7)
Caudate/Insula	22	L	7.69	(−21, 20, 22)
			7.34	(−15, 26, 25)
			7.27	(−21, 26, 10)

*Note: Table only includes significant gray matter clusters*.

Since there was no difference regarding the ethnic background between minor vs. major or heterozygous allelic groups (see above) the BOLD signal group differences cannot be attributed to differences in ethnic background. However, it cannot be excluded at this stage that differences in BOLD between heterozygous and homozygous major groups (see Figures 3B,C) may depend on minor differences in ethnic composition of the groups (see above).

#### Stop Signal Task

**Figure 4:**
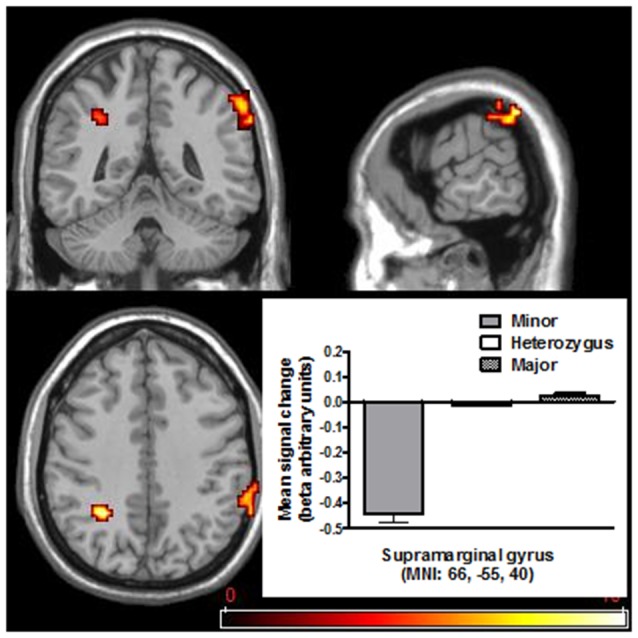
**Activity enhancement or reduction associated with “stop success” vs. “go success” contrast (SST contrast) in the group of homozygous minor, heterozygous and homozygous major for the SNP rs2044081.** Data are presented in mean ± SEM.

There was a difference between genotypes in BOLD response found in the right hemisphere supramarginal gyrus (*F*_(2,516)_ = 12.75, *p* < 0.005; see Figure 4), right hemisphere lingual (*F*_(2,516)_ = 10.93, *p* < 0.005) and left hemisphere Inferior parietal Gyrus (*F*_(2,516)_ = 11.32, *p* < 0.005), indicating a reduced BOLD response in the minor genotype (see Table 3 for details in the brain areas).

**Table 3:** **Whole brain magnitude related *F* scores and MNI coordinates of response peak for main effect of allelic group during SST task**.

Region	Cluster	L/R	*F*	MNI coord (*x, y, z*)
Supramarginal gyrus	68	R	12.75	(66, −55, 40)
			7.95	(66, −46, 43)
			7.52	(66, −49, 34)
Inferior parietal	27	L	11.32	(−27, −52, 34)
Lingual	23	R	10.93	(18, −70, −11)
			5.65	(18, −58, −8)

*Note: Table only includes significant gray matter clusters*.

Differences in the supramarginal gyrus reflected a significantly reduced brain response in the minor compared to heterozygous and major allelic group (*t*_(134)_ = −4.46, *p* < 0.001 and *t*_(395)_ = −2.63, *p* < 0.001 respectively).

Differences in the lingual gyrus reflected a significantly reduced BOLD response in the minor compared to heterozygous and major allelic group (*t*_(134)_ = −4.72, *p* < 0.001 and *t*_(395)_ = −4.33, *p* < 0.001 respectively) whereas differences in the parietal gyrus reflected an increased response in the major compared to heterozygous allelic group (*t*_(509)_ = −4.15, *p* < 0.001).

### Regression Analysis

#### Monetary Incentive Delay

The bold response associated with MID contrast in IFG was positively associated with the probability of responding on high win vs. no win trials (MID-diff; contrast value 3.04, FWE 0.001). No significant correlations with behavior were found for the other clusters; regression models with audit score did not result in any significant associations with changes in the BOLD signal.

#### Stop Signal Reaction Time

No significant correlations were found.

## Discussion

The relevance of *GABRB1* in determining alcohol preference in man is suggested by a recent study showing an association of between the intergenic SNP rs2044081 SNP in *GABRB1* with AD (McCabe et al., 2017). Previous studies have demonstrated significant allelic association between the risk for AD and both *GABRA2* and *GABRB1* polymorphisms in humans (Parsian and Zhang, 1999; Sun et al., 1999; Porjesz et al., 2002; Song et al., 2003; Edenberg et al., 2004, 2005).

It is unclear how variations in a non-coding region of *GABRB1* contribute to either altered susceptibility to AD, or to altered brain function during the performance of psychometric tasks. One possibility is that the intronic variation contributes to efficiency of expression of the gene, as has been suggested for intronic SNPs of *GABRA2* associated with AD (Lieberman et al., 2015). Although we have previously reported that two independent mutations of mouse *Gabrb1* lead to enhanced ethanol consumption in mice (Anstee et al., 2013), it is highly unlikely that variations in rs2044081 mimic such an effect. The mouse mutant studies implicating β1 found that the mutations of the gene giving rise to increased alcohol intake did so by allowing spontaneous chloride flux through affected GABA_A_ receptors. We do not know that this effect is unique to β1-containing receptors, and it is likely that homologous mutations in other members of the β subunit family would have similar consequences for channel gating, though whether they would have similar behavioral effects is unknown. Thus the mouse studies provide only partial evidence of a role of β1-containing GABA_A_ receptors in the control of alcohol drinking.

Second, in the human study, the rs2044081 SNP is located in a non-coding region of the gene, and may reflect linkage with a nearby chromosomal region, rather than direct effects on β1 itself. Nearby genes include *GABRA2*, for which a significant body of work suggests a link to alcohol use disorder. Nevertheless, taken together, the mouse and human studies refocus attention on the GABA_A_ β1 subunit as a potential contributor to addictive phenotypes.

Rather than the association between β1 SNP variants and alcohol abuse reflecting altered sensitivity of the receptor to ethanol, the genetic variations may give rise to behavioral traits such as altered reward sensitivity or impulsivity that predispose to loss of control over excessive drug use. However, our data did not find a relationship to alcohol use history in this population of adolescents. Variations in GABA_A_ receptors play a significant role in impulsivity traits related to drug (and especially alcohol) misuse, in particular when associated with early life stress (Dick et al., 2010, 2013; Villafuerte et al., 2012, 2013; for a review see Stephens et al., 2017). Importantly, in our sample, a LEQ did not reveal any differences across the allelic groups.

Nevertheless, contrary to our expectations, within the adolescent sample, the rs2044081 allele was not associated with an impulsive or reward-sensitivity phenotype as measured by SST and MID-Diff performance. Importantly, however, both SST and MID task performance produced brain activity changes, which differed across genotypes. Thus, in SST, significant differences in brain response during performance were seen in areas associated with inhibitory control and attentional processing. According to expectation, a reduced brain response was seen in the homozygous minor genotype compared to heterozygous and homozygous major genotype in regions associated with inhibitory control (e.g., right supramarginal gyrus) and visual working memory (lingual gyrus) and compared to homozygous major in regions associated with attentional monitoring (e.g., inferior parietal cortex). The altered brain responses in areas associated with task performance despite unaltered performance may indicate that in these individuals, at this developmental stage, compensatory changes in brain activity may serve to overcome potential deficits in performance. Alternatively, the measure of the brain response may simply be more sensitive than the measure of behavior, so that the behavioral changes are not detected.

Inferior parietal cortex activation has previously been found bilaterally during SST performance by Rubia et al. (2001), who concluded that this effect was due to movement-related visuospatial attentional demands which may be higher in inhibition tasks. Activations in Parietal and Temporal cortices areas have also been demonstrated previously during SST performance (Nikolaou et al., 2013a). Interestingly, alcohol given acutely reduces activation of inferior temporal cortex during successful stops in SST (Nikolaou et al., 2013a).

There was no significant difference between allelic groups regarding performance in the MID task. However, that differences in BOLD response of left IFG during performance were seen across the allelic groups suggests that greater activation was required in the homozygous minor group compared to other two genotypes, for equal level of performance of the task. Apart from its regulatory function in inhibiting pre-potent responses (Menon et al., 2001; Aron et al., 2003a,b; Picton et al., 2007; Nikolaou et al., 2013b), IFG has also been associated with the detection of salient cues carrying emotionally important information (Hampshire et al., 2009, 2010). Interestingly, IFG responses were associated with the probability of responding on high win vs. no win trials in the MID task.

Caudate/insula were also found to be more activated during MID performance in the homozygous minor group compared to heterozygous and homozygous major genotype. These areas are involved in the cognitive and emotional processing of reward (striatum e.g., O’Doherty et al., 2002; insula e.g., Tobler et al., 2006), and we have also shown these areas (striatum and insula) to be activated in another reward anticipation measure, the incentive conflict task (Duka et al., 2011). Knutson et al. (2000) have also shown increased putamen activation during performance of the MID task. The putamen is rich in dopaminergic terminals and along with the caudate makes up the dorsal striatum, an area heavily implicated in supporting motivational behavior associated with reward (Knutson et al., 2000). Increased BOLD responses in caudate in the homozygous minor group over the other groups may indicate greater sensitivity to reward, leading in turn to increased IFG activity (seen also in the homozygous minor group), presumably because participants were holding the outcome of the MID predictive cues in working memory (Krawczyk et al., 2007). This suggestion may be supported by the fact that correlations showed that the higher the response in the IFG, the higher the anticipation response difference between large and small reward.

Increased brain responses during MID were also seen for the homozygous minor allelic group relative to the other two genotypes in the ITG. This area has been associated with visual perception and recognition (Greem and Proffitt, 2001), perhaps suggesting that altered function in this area may contribute to changes in cue recognition important in initiating the reward anticipatory response.

Although an association with rs2044081 in *GABRB1* and AD has been identified in predominantly middle-aged adults (McCabe et al., 2017), we found no significant difference in the overall AUDIT score or on alcohol drinking habits in our sample of adolescent participants. However, this is not surprising as the adolescent participants may be yet to develop severe alcohol-related problems.

A strength of the present study is the sample size and cultural diversity of the adolescent group. The generalizability is supported by the fact that testing center was never a significant covariate for SST and MID performance indicating there was no effect of country on the results. A potential weakness of the study is the measure of impulsivity. The SST is an impulsive action task which directly measures motor inhibition, while the MID is usually interpreted as a measure of reward anticipation, rather than impulsivity (but see Peña-Oliver et al., 2016).

In conclusion, the present study finds in adolescents that variations in *GABRB1* are associated with altered brain responses in regions implicated in reward processing and behavioral control during performance of the MID, and SST respectively. While we found no evidence to directly implicate these variations of *GABRB1* as risk factors for impulsivity and reward sensitivity phenotypes, successful performance in these tasks may reflect altered function in certain brain regions in adolescents.

However, whether these individuals will ultimately show a higher incidence of addictions will reveal itself in follow up studies over the next 20 years. The current article suggests that it will be worthwhile investigating the *GABRB1* gene in these follow-up studies.

## Author Contributions

All authors listed have made substantial, direct and intellectual contribution to the work, and approved it for publication.TD and DNS wrote the manuscript which was approved by coauthors.

## Conflict of Interest Statement

The authors declare that the research was conducted in the absence of any commercial or financial relationships that could be construed as a potential conflict of interest.

## Footnotes

^1^https://www2.bc.edu/sd-slotnick/scripts.htm

^2^http://marsbar.sourceforge.net/
